# Empowerment in prevention: a qualitative inquiry into Black girl-centred strategies for reducing HIV/STI and drug misuse risk

**DOI:** 10.1080/26410397.2024.2444728

**Published:** 2025-01-29

**Authors:** Ijeoma Opara, Emmanuella Asabor, Jaleah Rutledge, Jasmin R Brooks Stephens, Sandy Cayo, Beatriz Duran-Becerra, Jasmine Abrams

**Affiliations:** a Associate Professor, Department of Social & Behavioral Sciences, Yale School of Public Health, New Haven, CT, USA.; b PhD Candidate, Yale School of Public Health, New Haven, CT, USA; Medical School Student, Yale School of Medicine, New Haven, CT, USA; c Postdoctoral Fellow, Yale School of Public Health, New Haven, CT, USA; d Postdoctoral Fellow, Department of Psychiatry, Massachusetts General Hospital, Boston, MA, USA; e Assistant Professor, Yale School of Nursing, New Haven, CT, USA; f Research Staff, Yale School of Public Health, New Haven, CT, USA; g Research Scientist, Yale School of Public Health, New Haven, CT, USA

**Keywords:** empowerment, HIV prevention, drug misuse prevention, Black adolescent girls, focus group methodology

## Abstract

Black girls in the United States are disproportionately diagnosed with sexually transmitted infections (STIs), which can increase the risk of contracting HIV (human immunodeficiency virus), compared to adolescent girls of other races. Therefore, this study was designed to understand barriers to impactful HIV/STI and substance use prevention programmes for Black girls. Data was collected between October 2021 and June 2022 from twelve focus groups which included (*N* = 62) participants who identified as Black and female between the ages of 13 and 18 years old. Using intersectionality theory as a guiding framework, the data were analysed thematically. The analysis revealed three themes: (1) need for parent–child discussions on sex; (2) stigma in schools; (3) suggestions for Black girl-centred prevention programming. Participants described minimal discussion about sex in their household as well as STI/HIV and substance abuse stigma as barriers to impactful sex education. They also indicated that current substance use and STI/HIV prevention interventions are not tailored to the experience of Black girls and offered suggestions for improvement. In sum, participants pointed out several barriers to existing sex education and substance abuse prevention programmes. Findings revealed that HIV and drug use prevention information and knowledge may be best received from older peers in small private settings that can allow for intimate discussion and sharing of reliable information on HIV education, aetiology, and safer sexual practices. In addition, the study findings support the need to develop a racial and gender-specific prevention programming that fosters peer relationships, social support, and empowerment-based principles.

## Plain language summary

Black girls in the United States face a higher risk of sexually transmitted infections (STIs), including HIV, compared to girls of other races. This study aimed to explore the barriers that make it difficult for HIV/STI and substance use prevention programmes to effectively reach and help Black girls. We conducted twelve focus groups with 62 Black girls aged 13–18 to gather their thoughts and experiences. We used intersectionality theory to understand how different aspects of their identities, like race and gender, affect their lives and access to health information.

The study revealed three key findings:

1. ***Need for Parent–Child Conversations*:** *Many girls felt that their parents did not talk to them enough about sex, which left them uninformed.*
2. ***Stigma in Schools*:** *The girls reported that stigma around HIV, STIs, and substance use in schools made it harder for them to access accurate information and support.*
3. ***Better Programmes for Black Girls*:** *The girls suggested that current prevention programmes are not designed with their unique experiences in mind. They want programmes that are more tailored to their needs, with opportunities for open discussions in small groups led by older peers.*

In conclusion, this study highlights the importance of creating prevention programmes specifically for Black girls. These programmes should encourage peer support, empower the girls, and provide safe spaces for them to learn and talk about HIV, STIs, and substance use.

## Résumé

Aux États-Unis, un diagnostic d’infection sexuellement transmissible (IST), qui peut augmenter le risque de contracter le VIH (virus de l’immunodéficience humaine), est posé de manière disproportionnée chez les jeunes filles noires, par rapport aux adolescentes d’autres races. En conséquence, cette étude avait pour objet de comprendre les obstacles à des programmes opérants de prévention du VIH/des IST et de l’abus de drogue pour les jeunes Noires. Les données ont été recueillies auprès de 12 groupes de discussion entre octobre 2021 et juin 2022, comprenant des participantes (*n* = 62) qui s’identifiaient comme Noires et de sexe féminin, âgées de 13 à 18 ans. Les données ont été analysées thématiquement en utilisant la théorie de l’intersectionnalité comme cadre directeur. L’analyse a révélé trois thèmes: (1) la nécessité de discussions parents-enfants sur la sexualité; (2) la stigmatisation à l’école; (3) des suggestions pour les programmes de prévention centrés sur les jeunes Noires. Les participantes ont cité les discussions minimes sur la sexualité dans leur famille ainsi que la stigmatisation des IST/du VIH et de l’abus de drogue comme obstacles à une éducation sexuelle efficace. Elles ont également indiqué que les interventions actuelles de prévention de la consommation de drogue et des IST/du VIH ne sont pas adaptées à l’expérience des adolescentes noires et ont proposé des suggestions d’amélioration. En résumé, les participantes ont signalé plusieurs obstacles aux programmes existants d’éducation sexuelle et de prévention de la toxicomanie. Les résultats ont révélé que les informations et les connaissances sur la prévention du VIH et de la toxicomanie peuvent être mieux reçues de pairs plus âgés dans de petits environnements privés qui permettent des discussions en toute intimité et le partage d’informations dignes de foi autour de l’éducation sur le VIH, de l’étiologie et des pratiques sexuelles à moindre risque. De plus, les conclusions de l’étude étayent la nécessité de mettre au point un programme de prévention spécifique à la race et au sexe qui favorise les relations entre pairs, le soutien social et les principes fondés sur l’autonomisation.

## Resumen

En Estados Unidos, las adolescentes Negras reciben de manera desproporcionada un diagnóstico de infecciones de transmisión sexual (ITS), que puede aumentar el riesgo de contraer el VIH (virus de inmunodeficiencia humana), en comparación con las adolescentes de otras razas. Por esta razón, se diseñó este estudio para entender las barreras a programas impactantes de prevención de VIH/ITS y de toxicomanía dirigidos a adolescentes Negras. Entre octubre de 2021 y junio de 2022, se recolectaron datos de doce grupos focales integrados por participantes (*N* = 62) que se identificaron como Negras y de género femenino entre las edades de 13 y 18. Utilizando la teoría de la interseccionalidad como un marco rector, se analizaron los datos temáticamente. El análisis reveló tres temas: (1) la necesidad de entablar conversaciones entre padres e hijas sobre el sexo; (2) el estigma en las escuelas; (3) sugerencias de programas de prevención centrados en las adolescentes Negras. Las participantes describieron mínima conversación sobre el sexo en su hogar, así como el estigma de ITS/VIH y de la toxicomanía como barreras a una educación sexual impactante. Indicaron que las intervenciones actuales de prevención de toxicomanía y de ITS/VIH no están adaptadas a la experiencia de las adolescentes Negras y ofrecer sugerencias para mejorar. En resumen, las participantes señalaron varias barreras a los programas actuales de educación sexual y prevención de toxicomanía. Los hallazgos revelaron que la mejor manera de recibir información y conocimientos sobre la prevención del VIH y la toxicomanía es por medio de pares mayores en entornos privados más pequeños que se presten para conversaciones más íntimas y para compartir información fidedigna sobre educación en VIH, etiología y prácticas sexuales más seguras. Además, los hallazgos del estudio corroboran la necesidad de crear programas de prevención dirigidos a razas y géneros específicos, que fomenten relaciones entre pares, apoyo social y principios basados en empoderamiento.

## Introduction

Among adolescent girls in the United States (US), there are significant racial disparities in HIV and STI diagnoses. For example, Black girls in the US between the ages of 15 and 19 years had 4.5 times the rate of chlamydia compared to White girls.^1^ In addition, the rate of reported gonorrhoea cases among Black adolescent girls was 8.8 times the rate among White adolescent girls.^1^ Research has indicated that there are multiple contextual and interpersonal factors that can increase sexual risk behaviour, which can lead to transmission of sexually transmitted infection during adolescence, such as peer and familial substance use,^2^ neighbourhood disorganisation,^3^ alcohol-outlet density,^4^ gender-based violence/victimisation,^5^ and mental illness.^6^ These risk factors can disproportionately affect Black girls, thereby impacting educational outcomes,^7^ mental health,^8^ unintended pregnancy, rates of sexually transmitted infections/HIV,^9^ and other aspects of physical health.^10,11^ Opara et al^12^ conducted a systematic review of HIV/STI and drug use prevention interventions that have been delivered to Black adolescent girls and found that of 15 interventions, only four of them were culturally adapted to cater to Black girls while only one intervention focused on both HIV/STI and drug use prevention for Black girls. Given the rising rates of substance misuse among this population, it is essential that prevention interventions to promote healthy behaviours be catered and more accessible to Black girls in the US.

Despite these findings, many HIV and STI and drug use prevention approaches targeted towards Black girls use a deficit framework by focusing on individual-level risk factors.^12^ This approach fails to address important socio-cultural and structural factors that may contribute to racial sexual health disparities.^12,13^ Considering that sexual health is experienced within individual beliefs, values, and relationships, it is imperative to consider socio-cultural and structural factors including gendered racism, historical sexual scripts, and access to health education and resources that directly and indirectly influence risky behaviours.^12,14^

Given the historical and socio-cultural context of highly stereotyped Black sexuality, sex is often associated with stigma and shame in the Black American community. The sexual socialisation process Black girls undergo with parents emphasises abstinence, gendered sexual roles, and few sex-positive messages, leaving girls with a dearth of information about healthy relationships and interactions, their anatomy and bodily concerns, or sexual education from their families.^15,16^ This often leads to Black girls hiding sexual behaviours to avoid punishment for their sexual exploration.^17^ Addressing sexual health disparities requires society to address the stigma surrounding sexual exploration of Black adolescent girls and guide them in developing a healthy sense of self in the context of their sexuality.^16,18^

Parents and other family members play an important role in the sexual socialisation of adolescents through communicating their expectations regarding sexual activity and modelling risk reduction strategies and behaviours.^19^ Among Black girls, social support is a protective factor against substance use and sexual risk behaviours.^10,15,19–21^ Specifically, relationships between Black girls and their mothers are unique, serving as a source of empowerment and racial socialisation for Black girls that can be critical to their development and navigation of high-risk behaviours.^22^ Further, strong mother–daughter relationships are associated with high self-esteem, strong ethnic identity, adaptive coping strategies, and decreased drug use among adolescent girls.^19,23–25^

### Purpose of study

Using the voices of Black girls, this study aims to uncover prevention strategies that Black girls identify as most important in HIV/STI and drug use prevention programming. We approached this qualitative study using an Intersectionality framework^26^ to ensure that the analysis and findings comprehensively represented Black girls’ experiences by considering their unique social identities and experiences, specifically at the intersection of racism and sexism. This is essential because Black girls’ social positioning as a function of the multiple marginalised identities they embody often makes them susceptible to a myriad of historical, structural, and cultural forms of discrimination, such as racism^27,28^ and classism,^29^ that shape their experiences with sex, STIs, HIV, and substance abuse.

## Methods

### Participants

The study is a part of a larger study focused on developing an HIV and drug use prevention programme for Black girls, funded by the National Institutes of Health^12^ and approved by the Institutional Review Board at Yale University in 15 June 2020 (IRB #2000026660). Due to the sensitivity of the topic, parent written consent was waived and only verbal assent from youth participants was required. To be eligible to be a part of this study, all participants had to identify as female, Black, and be between the ages of 13 and 18 years old. Recruitment included word of mouth from youth-serving organisations and posting of a flyer on social media platforms such as Twitter and Instagram. All interested individuals (1215) first completed an eligibility form then the study project manager individually contacted potential participants to confirm their age, race, and gender. During this process, 1100 individuals were excluded from participating in the study for not meeting the eligibility criteria which included mostly not being within the target age group or race (not identifying as Black). Eligible individuals were invited to participate in the study via email and a study staff member called each eligible individual to conduct a screening interview. Once eligible participants met screening criteria and confirmed interest in participation, they were given the option to participate in available focus group times.

All of the focus groups were led by research assistants who identified as Black women. The first focus group was conducted in October 2021 and recruitment ended in June 2022 when the last focus group took place. This was done due to the importance of racial and gender matching in the data collection phase, which can help create a welcoming research environment and encourage active participation.^30^ All youth participants verbally assented to participate at the start of each focus group and were given an information sheet about the study. Information sheets were mailed to all participants under the age of 18 years old and to their parents/guardians to inform them about the study. The focus group interviews were conducted within nine months of the initial posting of the recruitment ad. Black girls (*N* = 62) between the ages of 13 and 18 (*M* = 15.6 years, *SD* = 1.50) participated in the focus groups. In total, 12 focus groups were conducted; 4 groups were online, 4 groups were in-person at a youth-serving organisation, and 4 groups were in a closed, private space at a high school. The in-person groups (eight) were held in two northeastern New Jersey urban cities that the study principal investigator has strong connections to. Girls that attended the online groups were asked verbally if they were alone and in a closed-door room before the interviews started. All of the girls in the online group responded that they were alone.

### Researcher’s positionality

The research team collectively consists of all women of colour who are dedicated to improving the lives of Black girls and work under a larger NIH-funded study that centres on engaging with communities of colour and highlighting the strengths of Black girls. While we share similar race/ethnic attributes with the participants in the study, we acknowledge that we are not experts in their lived realities as Black girls. All of the authors of the study were involved in either data collection or data analysis with the exception of the last author who takes an objective stance as an expert on intersectional qualitative research and provided guidance on the interpretation of the findings to ensure that analysis and interpretation of the findings were consistent with using intersectionality as a framework.^31^

### Data collection

We conducted 12 focus groups to enhance rigour and ensure a diverse representation of participants and their perspectives.^32^ The focus groups were audio-recorded through the university’s secured Zoom video conferencing software. A phenomenological framework^32^ was used to guide the development of semi-structured focus group interview questions. The focus group guide (see Appendix A1) was developed iteratively by the principal investigator and incorporated feedback from her research team and community partners. Facilitators guided the discussion following the semi-structured focus group guide. Interviews were then transcribed verbatim. The focus groups ranged from 90 to 120 minutes and each focus group consisted of six to eight participants. Each group had at least one co-facilitator who took notes and memos. At the end of each focus group, participants were either emailed a $30 gift card or were given a gift card in person.

### Data coding and analysis

Audio recordings were transcribed by a professional transcription service and verified for accuracy by the research team. The research team which consisted of four research assistants coded all transcripts utilising an Intersectionality approach to qualitative data.^31^ Data from the interviews were first analysed by the research team using open coding, whereby concepts were identified and labelled as they emerged from the data and across the focus groups.^33^ The research team read and re-read all the transcripts and developed initial codes in isolation. During weekly research team meetings, the initial codes were reviewed and discussed until all team members agreed on the coding and no discrepancies remained. The team agreed on the process of coding before the analysis occurred. All coding was conducted by hand and were then imported into NVivo, version 13.

### Ethics approval and informed consent

The study protocol, consent forms, interview guides, and all participant-related materials were approved by the Yale University Institutional Review Board (2000026660) on 15 June 2020. The protocol was modified to include a larger sample of adolescent girls and approved on 17 March 2022 (2000026660). Due to the sensitivity of the research questions, we requested waived written parent consent for youth participants which was approved by the Institutional Review Board. All youth who participated in the study received a youth information sheet and their parents/guardians will receive a parent information sheet. All youth gave verbal consent before participating in the focus groups and were told that they could leave the focus group at any time. The youth and parent/guardian information sheets included the study principal investigator’s contact information and participants were encouraged to contact the principal investigator if they had any questions pertaining to their involvement in the study.

## Results

During the data analysis, major themes arose from Black girls across all twelve focus groups about their views pertaining to sexual health education. Stigma was an overarching theme when girls were asked to discuss their views of HIV, STI and drug use education. Girls in the study agreed that they needed more information about prevention strategies and provided suggestions on what kinds of messaging would be most effective for them. Three major themes arose from the data using thematic analysis: (1) Need for parent–child discussions on sex; (2) Stigma in schools; (3) Suggestions for Black girl-centred prevention programming.

### Theme 1: need for parent–child discussions on sex

When discussing sexual health education specifically, girls felt that they did not receive enough and believed that most should come from their households. They acknowledged that there is discomfort in talking to their parents about sex, however, the majority of participants in all focus groups collectively agreed that they wished it was not awkward as their parents would most likely be the best source of quality information. One of the participants mentioned her views, acknowledging that although she is in high school, she has not received sexual education in her school yet:

*“I feel like with Black girls especially, I haven’t had sexual education yet, but I feel like the conversation isn’t had in a lot of households, my mother never had that conversation with me, I kinda just figured it out. This one right here {referring to a friend in the focus group}, told me everything about sex. I didn’t know anything about sex until about 11, 12 years old. I didn’t know anything. My mother never really had the conversation.”* (17-year-old participant)

Another participant in the same focus group added,

*“To add on to what she said, okay, when you get that talk, I feel your parents should tell you the effects of having sex and stuff like that. They really should, because that could prevent you from doing it because you think that you don’t want to do it because of the dangers and risk.”* (16-year-old participant)

Other participants discussed similar views of lack of education in general and the need for families to have honest conversations about sex and drugs. The girls also felt that there were obvious racial differences in how Black girls versus White girls talk to their parents about sex:

*“I think in some White families, when their kids start having sex, they’re open with their parents and they’re open with their children and they talk about it. But Black girls, their parents are, and I’m not going to say all parents, but I know my parents, they never talk about the sex talk. They always talk about, ‘Oh, you can’t watch this,’ or, ‘You can’t see this,’ or, ‘You can’t dress like this.’”* (14-year-old participant)

Other girls who mentioned that their parents have talked to them about sex and drugs, felt that even when they do have this conversation, it feels that it comes from a judgmental place, making them uncomfortable and then results in them learning about sex and drugs from other sources.

*“When they do start, your parents are judging you and you feel like you are not supposed to do it based on how they treat you about it. I feel not a lot of girls understand what it is and they got to learn from watching TV or their friends. That’s why when actually parents find out, both sides are hurt because they didn’t get the correct talks for stuff and what they go through.”* (15-year-old participant)

Participants across all the focus groups mentioned that discussions around HIV, sex, and sexual health were highly stigmatised topics and often not discussed. One participant, who identified as a Black immigrant, reflected on her experiences in the way people from her home country discussed HIV:

*“It’s only women, especially women who sleep around and always catch HIV or that person is always sleeping around that’s the one who’s going to get HIV … HIV – it was like the worst thing you can get in your life is HIV that was how I learned about it.”* (17-year-old participant)

Most of the participants mentioned that they have only learned about HIV prevention within the context of gay men and have not received gender-specific messages pertaining to HIV prevention that are catered to Black girls.

Participants were also aware that stigma surrounding sex intersects with gender roles and objectification of girls and women. One participant explained that there are double standards when it comes to sex, which impact conversations about sex:

*“It’s sort of a taboo topic to be honest because my mom never sat me down and had a conversation with me about sex, I don’t think any of us has. Again, it just goes back to that whole stigma thing because for some reason there is a stigma around sex. However, girls are shamed for having sex while boys are praised for having sex. Girls are compared to objects daily … they shame us for even wanting sex.”* (16-year-old participant)

Overall, girls in the study were aware of the importance of families talking about sex to them but acknowledged that these conversations often did not occur in their families. They attributed this lack of discussion to parental protectiveness, gender role views of Black girls, and overall stigma of sexually transmitted infections and the act of sex.

### Theme 2: stigma in the classroom

A majority of participants indicated learning about sexual health at school. However, they explained that the stigma surrounding sex limits the topics and resources they learn about in school. Another participant commented on not only how much she felt the school talked about HIV/AIDS but the lack of education that they generally receive on sexuality and pregnancy.

One of the ways that participants described how stigma affected their ability to have important conversations is the awkwardness that teachers have related to discussing sex. They highlighted that teachers often steer away from discussing certain sexual health topics in the classroom. One participant noted:

*“ … at my school, we had health one year and we learned about sexual health for one unit and it’s just like interesting to see how … you just kind of like skim over it, because, like oh it’s kind of uncomfortable to talk about and they’d … don’t want to … deal with … awkward teenagers talking about you know sexual health or like kids not being able to take it seriously. But then it’s like they … expect the kids to know it when a lot of them … maybe don’t … .”* (16-year-old participant)

Participants emphasised how that discomfort impacts their ability to have conversations about sex in their health classrooms and the extent to which students were receptive to the topics being taught. One participant reflected on the collective discomfort she felt with her classmates when learning about sexual health at school:

*“There’s this like weird discomfort and we end up admitting it in like jokes. If we talk about it [sex], or if the word sex appears on like a TV screen or it appears on the whiteboard, our first initial reaction is we’re confused about it so we just respond negative towards it. For example, people say ew, people start laughing and we could be so immature about it … Just having something where people can come and talk about it, and just not having to take the class, not having to be uncomfortable, not having to be in that situation.”* (13-year-old participant).

The stigma and discomfort of talking about HIV, STIs and sex not only led to avoidance of certain sexual health topics at school, but also to dismissal of students’ sexual health needs. For example, one participant recounted a time when the sexual health teacher at school avoided talking about chlamydia with her students despite being aware of a chlamydia outbreak at school.

Girls discussed having to look for other sources of information such as online videos, friends, and social media when certain sexual health topics are ignored or underexplained:

*“When I started to learn about this was never in an actual formal setting like it was never like someone sat me down. It was more of a common knowledge between friends. I had friends who were you know having sex and all that. Because you know in [my home country] the biggest taboo is HIV, having a baby out of wedlock, so it was more of those things we talked about … but we never actually talked about ‘it.’ It was more like ‘whenever you have sex you use condoms, birth control pills … .’”* (17-year-old participant)

Overall, participants felt that the stigma regarding sexual health and STIs had a negative impact on girls’ sexual health knowledge and their ability to protect themselves. Many voiced the need to address sexual health stigma as well as wanting to have more honest conversations on sexual health in a more comfortable environment.

In addition to wanting more thorough information on safe sex and the consequences of unsafe sex, girls in the study identified the limited accessibility of sexual health resources, such as condoms and STI testing, as significant determinants of sexual health for Black girls. One participant shared how lack of access to resources impacted a friend:

*“One of my friends has never had protected sex and she’s got a boyfriend for three years. I had to buy her like a pregnancy test through my mom … because our school doesn’t give free condoms, and neither one of them wanted to ask their parents, so they decided to just risk it that way … So I think that if our school just allocated like resources to take kids on like bus trips to go to Planned Parenthood … to get these like book these appointments with them … It can make things so much better … .”* (16-year-old participant)

Another participant mentioned the hyperfocus on HIV/AIDS while overlooking other important sexual health topics such as teen pregnancy, gender and sexual identity, and reproductive rights.

*“I feel like our school really tries to put the pressure on people to talk about HIV and AIDS, which is very important to discuss, but doesn’t talk enough about teen pregnancy or like LGBT to sex or like not promoting abortions, but educating on what abortion is, how to get access to these things, passing out condoms … .”* (16-year-old participant)

Highlighting the need for intersectional approaches to research and intervention work, this participant, described the necessity approaching in sexual health promotion among Black girls through a more expansive and comprehensive lens – moving beyond a mere focus on disease prevention and examining other experiences with and consequences of having sex.

When the interviewers asked participants how their communities should address HIV and STIs, many participants felt that, besides mentioning the topics and using scare tactics, their communities were not doing anything significant to address these issues. Although girls were able to recall information they had learned about HIV and STIs from various sources, many girls expressed a need for more information on these topics:

*“A lot of us are not informed about any of that these days and if we are informed it’s just really quick like a 5-minute video … But most of those times we don’t remember those 5-minute videos, we really need to be educated on the matter.”* (17-year-old participant)

This participant expressed her dissatisfaction with the extent to which sexual health information is discussed, thus highlighting gaps in existing sexual health information in her community.

### Theme 3: suggestions for Black girl-centred prevention programming

Girls in the study expressed that existing sexual health and substance use prevention interventions are not tailored to the specific experiences and needs of Black girls. Several sub-themes emerged around ways to centre Black girls in prevention programming.

#### Subtheme 1: young Black women as intervention facilitators

Sharing a racial and gender identity with programme facilitators was identified as critical factor for making interventions more relevant for Black girls. One participant identified a need for more culturally, gender-relevant conversations around sexual health:

*“We have a lot of white teachers that don’t reflect the diversity of our student population, and I feel like it just creates that disconnect between like our experiences and theirs. And, they may not acknowledge that or they may not want to acknowledge the fact that maybe we do have a lot less knowledge about like sexual health and things like that.”* (16-year-old participant)

This participant underscores how a lack of shared experiences between intervention facilitators and intervention participants can limit the type of information participants receive during an intervention programme. Across most focus groups, girls felt that including people who did not identify as Black women in the intervention delivery would reduce the effectiveness of the programme. Girls stated that having facilitators who could identify with programme participants would create a needed safe space for Black girls to freely talk about their shared experiences.

In addition to race and gender, age was identified as an important factor to consider when choosing intervention facilitators. Girls felt that programme participants would be most receptive if facilitators were teenagers or college-aged people. Participants perceived that older people would have “different views from teens now,” be judgmental, and share more traditional views such as “wait till after marriage” or “you have to please your man.”

When asked who should facilitate the intervention, another participant emphasised the importance of including people with lived experience in the intervention delivery:

*“Someone who had to sit through being bullied for being Black, or substance abuse, alcohol abuse or through peer pressure or anything that we mentioned. Because they have experience with that, they can tell us what not to do now, and they will understand it since they went through it.”* (17-year-old participant)

Girls in the study also expressed wanting the intervention content to be relatable and realistic to teenage girls, which they felt having younger Black women would be able to deliver – due to being closer to their ages. One participant called for more transparent substance use information:

*“I want them to tell the truth and I want them to be honest … If you’re going to tell me to stop doing something, tell me a good reason why. They said ‘because it’s bad for you’ … I want to know the real reason, tell me that it will cause me cancer, or I can get addicted … Don’t just give me one lame excuse. Don’t say I need to listen to you because I really don’t have to.”* (18-year-old participant)

Girls in the study felt that adults commonly relied on their position of authority to influence teenagers’ behaviours instead of providing them with honest information. It became apparent that girls highly valued having access to comprehensive sexual health and substance use information in order to make their own best decisions. Another participant added the importance of hearing different perspectives:

*“Nobody wants to go to a prevention [program] and look at their mother. When it’s younger [facilitators], you know they’ve been through it … but you got to have … different perspectives … You can’t have everybody saying, ‘oh my God, sex is bad for XYZ.’ Some got to say ‘No, I had a good experience here … but two years later I realised XYZ.’”* (17-year-old participant)

Among girls in the study, there was a collective feeling that the sexual health and substance use prevention programmes that they have been exposed to were not effective because they were not relevant to their unique experiences as Black girls. When facilitators asked participants what they wanted to see in a sexual health and substance use prevention programme, most girls mentioned facilitators who were close to their age, identified as Black women, and had experience in sexual health and/or substance use. Girls felt that having facilitators that could relate and understand their experiences would create a safe environment for Black girls to receive comprehensive and non-judgmental sexual health and substance use information.

#### Subtheme 2: moving beyond abstinence-based approaches

Additionally, many participants felt that promoting abstinence was an unrealistic goal for youth and called for promoting safer sex practices instead. One participant explained how abstinence-only education can be counterproductive:

*“I feel it’s [sex] similar to drugs that if people have decided they’re interested in this thing they’re going to find ways to do it and the harder you make it for them to do it in a safe way, the more dangerous it just continues to get, that if we make sex so stigmatised people will just begin to have unsafe sex to avoid the consequences of those actions.”* (16-year-old participant)

Another participant shared her thoughts abstinence-based programming:

*“I feel like the first thing that you should not do is preach abstinence. Because like if you just tell teenagers not to have sex, it’s just gonna make them want to have sex more. But if you tell them why they shouldn’t have sex and you go into detail on why they shouldn’t have sex … Or not shouldn’t have sex, but more like protect themselves if they do wanna have sex, you know.”* (15-year-old participant)

Overall, girls in the study identified abstinence-focused sex education as an ineffective approach for protecting girls against HIV and STIs. Participants acknowledged that it is inevitable that some youth will engage in sexual activities. Creating an environment where they can engage in safe sex should be the goal of sexual health interventions for this group. Participants also reported an association between abstinence-focused education and feeling as though adults were trying to control their actions. This feeling of control exacerbated trust issues with authority figures among teenagers experiencing a “rebellious phase.”

Many participants felt that teenagers go through a period in their development where they are forging their unique identity and often utilise opposition to make independent decisions. Therefore, commanding youth to not do something would only increase some youths’ desire to engage in the forbidden activity. Although participants felt that it was important to discuss the consequences of having sex, they also identified the lack of conversations about safer sex practices and limited access to sexual health resources as factors contributing to negative sexual health outcomes among Black girls. Moreover, participants expressed that since youth have their own views and desire autonomy, attempting to control their actions is an unrealistic goal for adults, especially when they are not always around to supervise their teenagers. Instead, girls in the study felt that adults had a critical role in creating a safe environment for teenagers by providing them with information and resources, such as condoms and STI testing, in case they decided to have sex.

In sum, girls in the current study revealed several critical factors that should inform HIV and STI prevention for Black girls. These included parent and child discussion regarding prevention, destigmatising sex education in the classroom, prevention programming that is gender- and race-specific, along with targeted messaging to help promote prevention practices. Moreover, these findings underscore the need to develop interventions that are specific to intersections of race and gender, discussing sexual health comprehensively, and the importance of peer-led interventions.

## Discussion

This qualitative study applied an intersectional framework to analyse how the experiences of Black girls in drug use prevention and sexual health education are shaped by the interplay of race, gender, and stigma. Intersectionality, as a theoretical framework, recognises that identities such as race, gender, and age do not exist in isolation but rather intersect to create unique experiences of privilege and oppression.^19,31^ For Black girls, this intersection amplifies the challenges they face in accessing relevant and effective drug use prevention and sexual health education, as they navigate societal stigma, racial stereotypes, and gendered expectations. In analysing the results, intersectionality theory guided the exploration of how race and gender intersect to influence the stigma surrounding substance use prevention and sexual health education. Specifically, results revealed that young Black girls experience the impact of stigma on their HIV and STI and substance use prevention education across their environments. They articulated awkwardness on the part of parents, teachers and other adult figures when discussing sex and safer sex practices to prevent STIs. Participants’ discussions revealed how traditional gender norms, often compounded by cultural, familial, and racial expectations, framed conversations (or the lack thereof) specifically about sex within their households The participants noted that their parents’ discomfort with discussing sexual health stemmed not only from general societal stigma but also from racialised narratives that frame Black girls as needing stricter moral policing compared to their White counterparts. Findings from the study found that girls wanted to hear from their parents about sexual health; however, they felt that the topic of sex was such a taboo that it could not be discussed. This is consistent with the literature on parent–child sexual health communication that acknowledges the importance of parents discussing sex with their children,^34,35^ and the expressed desire of children for these conversations.^36^ However, despite the advantages of effective parent–child communication about sex, this type of conversation was either uncomfortable or absent for many of the participants in this study. This indicates a gap between the recognised importance of parent–child communication about sex and its implementation. These racialised and gendered dynamics further compounded the challenges girls faced when seeking accurate and non-judgmental information about sexual health.

Among Black adolescent girls, effective social supports through family and parenting has been seen to be a strong protective factor against substance use, and engaging in sexual risk behaviours such as not using condoms.^10,15,19,21^ Parents and other family members play an important role in the sexual socialisation of adolescents through communicating their expectations regarding sexual activity and modelling their preferred risk reduction strategies and behaviour.^19^ Additionally, parents are in a unique position to monitor and influence the peers that their child interacts with, reducing exposure to peers who engage in risky behaviours.^19^ Specifically, mother–daughter relationships between Black adolescent girls and their mothers are unique, serving as a source of empowerment and racial socialisation for Black girls that can be critical to their development and navigation of high-risk behaviours.^15,24,37^ Further, strong mother–daughter relationships are associated with high self-esteem, strong ethnic identity, good coping strategies, and have been shown to predict drug use among adolescent girls.^15,22^ Although less studied, the literature shows that father–daughter relationships are associated with higher drug refusal self-efficacy when compared to mother–daughter relationships.^20^ Importantly, in both mother–daughter and father–daughter relationships, it is the quality of the parent–child relationship that is an important predictor of adolescent risk behaviour.^38^ Parents can rely on themselves to provide their child with accurate information about the risks and consequences of risky behaviours, challenge the normalisation of drug use in their environments (i.e. peer use, drug use in their neighbourhoods), and teach their child crucial skills needed to make both responsible and informed decisions about substance use and sexual behaviours.^19^ These interactions build resilience and resistance among Black adolescent girls, where they can critically analyse their environments and prevent susceptibility to common negative stereotypes about Black girls.^12,19^ As such, future research should focus on identifying strategies to effectively support the initiation and maintenance of open dialogue about sex between adolescent Black girls and their parents.

The second theme uncovered the nuances of discussing sex in school, where girls felt that it was even more stigmatised, which limited teachers’ ability to provide quality sexual health education. Using an intersectional lens, within the context of schools, the analysis considered how structural inequalities, including predominantly White teaching staff and abstinence-focused curricula, failed to address the unique needs of Black girls. Participants highlighted that the absence of culturally relevant education and the stigma-laden environment created barriers to discussing sex openly, leaving Black girls to rely on peers or online sources for critical information. Intersectionality allowed the analysis to foreground how these intersecting identities – being Black, female, and adolescent – placed participants at the margins of sexual health education, where their specific concerns were overlooked or dismissed. Participants expressed a desire to experience stigma-free sex education in a safe environment, which echoes existing literature.^39,40^ In addition, these findings point to challenges with delivering sex education in an effective and stigma-free manner. This is somewhat unsurprising as prior research has documented adolescents’ concerns about stigma and gender stereotype reinforcement in school-based sex education.^39,41^ Overall, stigma appears to remain an obstacle, hindering the types of transparent conversations young Black girls felt were necessary to protect and prepare them for safer sex. These findings align with existing evidence on stigma as a barrier to STI/HIV prevention.^42–44^ Prior literature has revealed discord between the needs of Black girls in terms of conversations about sex and the approach of trusted adults to those conversations.^45,46^ Our study expands on these findings by highlighting ways to address the discordance between what Black girls’ desire in a conversation about sex versus what they typically receive.

Girls in the study also articulated the need for relatable prevention programming. They emphasised the importance of facilitators with some degree of lived experience. Although there is some evidence of the effectiveness for racial congruence in patient/health provider relationships^47,48^ more research is needed to evaluate the effectiveness of race, age, and gender congruence for the implementation of prevention interventions for Black girls. The girls in the study expressed a preference for prevention programming facilitated by peers or other relatively young people that have common identities as them (e.g. race and gender). Therefore, our study findings join calls for more research on peer-led interventions to prevent substance use among youth.^49–51^ By centring Black girls’ voices, the analysis illuminated how prevention programming must address the compounded effects of these intersecting identities. As participants emphasise the importance of having young Black women facilitators, as an example, they acknowledge the importance of creating safe spaces for open dialogue with individuals that share their racial and gendered experiences. The girls in the study also expressed a need for moving beyond abstinence-only approaches, which they viewed as disconnected from their current realities and reflective of adult attempts to control their behaviour rather than empower them with knowledge and resources.

Currently, sex education varies across the US, with a lack of adherence to the recommended national sex education standards, which attempt to provide guidance on the essential content and skills needed for sex education to be effective among youth.^52^ These essential components include: (1) Consent and healthy relationships, (2) Anatomy and physiology, (3) Puberty and adolescent sexual development, (4) (a) Gender identity and expression, (b) Sexual orientation and identity, (5) Sexual health (i.e. pregnancy, STIs, HIV), and (6) Interpersonal violence.^52^ Contrary to the established recommendations, not all states in the US require a sex education curriculum be taught. Indeed, 29 states and the District of Columbia currently mandate sex education to be taught, and 16 states provide abstinence-only education, defined as curricula that “only include abstinence or emphasise abstinence as the main way to avoid pregnancy and sexually transmitted infections.”^53^ Evidence demonstrates that abstinence-only education is ineffective at significantly reducing targeted behaviours, including delayed onset of sexual activity, or lowering STI or teen pregnancy rates.^54,55^ Further, the way in which abstinence-only programmes are taught grossly underprepares students by withholding information regarding alternative forms of contraception, and may provide stigmatising information, ultimately depriving students of pertinent education to make informed and safe sexual health decisions.^54,55^ This is particularly alarming for Black girls, who may engage in risky sexual behaviours such as low condom use, multiple sexual partners, and using alcohol before sex, at disproportionately higher rates than their White and Hispanic counterparts.^56^ Additionally, Black girls in the US continue to have higher rates of teenage pregnancy, and diagnoses of STIs, putting this group in need of a more effective race- and gender-specific sex education curriculum.^1,56–60^

In comparison to abstinence-only programmes, a comprehensive sex education curriculum, which uses a holistic and sex-positive framework that emphasises empowerment, gender equality, and critical thinking, may be particularly effective for Black girls.^53^ Evaluations of comprehensive sex education curricula have shown them to be effective in fostering an appreciation of sexual diversity, improving knowledge, attitudes, perpetration and victimisation of dating and intimate partner violence, increased skills and attitudes for the development of healthy relationships, improvement in social and emotional learning, and increased media literacy regarding how media affect one’s sense of self and perception of norms.^61^ Additionally, comprehensive sex education curricula that include content acknowledging gender and power led to the exploration of gender stereotypes and power inequalities in intimate relationships.^62^ Considering the negative impact that gendered and racial stereotypes have on the self-esteem and sexual risk behaviours of Black girls, including gender and power content may add a culturally relevant component to sex education that improves its efficacy among this population.^63^

### Limitations

Although our study has several strengths, we acknowledge that our study has some limitations. First, our participants are not a representative sample of all Black adolescent girls in the US as a majority of the sample lived in urban Northeastern, New Jersey, United States. As a qualitative study, the findings are not intended to be statistically generalisable. However, the insights gained from this research may be transferable to other groups of Black girls in urban settings who face similar structural and socio-cultural dynamics. Transferability in qualitative research relies on the contextual richness of the data and the extent to which the findings resonate with similar populations. The experiences described by participants – such as the stigma surrounding sexual health discussions, the limited accessibility of culturally relevant programming, and the intersectional barriers shaped by race and gender – are challenges commonly reported among Black adolescent girls. These shared systemic and social realities may make the study’s findings applicable to other urban contexts where Black girls encounter comparable educational, familial, and societal dynamics. Future research could benefit from an exploration of gendered racist stereotypes among a broader sample of Black girls in the US. The second limitation concerns the use of focus groups as opposed to individual interviews. Although focus groups have several benefits within the context of sexual health research among adolescents (e.g. capturing group processes, peer support), it is possible that some participants may have been hesitant to fully disclose their experiences and perspectives within the group setting due to the sensitive nature of the topic. The third limitation is that although girls mentioned the importance of parent–child communication in prevention, we did not explore what type of messaging would be beneficial from parents to daughter. As such, there is a need for future research to establish specific family processes and communication strategies that would further improve HIV/STI and drug use prevention education for Black girls.

### Implications

This study carries important implications for practice. First, the findings underscore a critical desire for more parent–child conversations on sex. The participants provided important insights regarding the absence of conversations and a general discomfort regarding conversations about sex with their parents. These results point to a greater need for implementing culturally tailored interventions designed to enhance parent–child communication about sex. In considering ways to best address the sex education needs of Black girls, participants expressed ideas most aligned with a comprehensive sex education approach (see Discussion).

Belgrave^64^ and Opara et al^15^ have highlighted the importance of cultural attributes in the design and implementation of HIV/STI and drug use prevention programmes for Black girls. They propose the integration of cultural elements such as ethnic identity, social support, community empowerment and gender roles as a strategy for developing effective HIV prevention interventions for Black girls.^12,15^ Echoing their recommendations, it is imperative that substance abuse and HIV prevention interventions for Black girls attend to the intersectional experiences of Black girls by attending to and including unique cultural elements.

## Conclusion

This study provides critical insight into enhancing the development and implementation of substance abuse and HIV/STI prevention programmes for Black girls in the United States. Findings from our study suggest that some of the best ways to engage Black girls in HIV, STI, and drug use prevention include parent and child discussion regarding prevention, destigmatising sex education in the classroom, and prevention programming that are culturally tailored. Our findings collectively propose that a comprehensive approach that supports prevention education for Black girls in the home and in schools. This approach should incorporate more than abstinence-only messaging and also focus on creating safe spaces for Black girls to learn and build connection with others including peers and family. We recommend the development of interventions that go beyond the individual level and address systemic racism and sexism which contribute to how Black adolescent girls view themselves and are treated in society. We encourage researchers to incorporate various levels of engagement in prevention interventions to eliminate stigma in discussions among teachers and also parents/families with Black girls.

## Supplementary Material

Appendix A1. Focus Group Protocol.

## Provenance statement

This article was not commissioned and has undergone external review.
