# Supplementary material for: Empowerment in prevention: a qualitative inquiry into Black girl-centred strategies for reducing HIV/STI and drug misuse risk
Source: Sex Reprod Health Matters. 2025 Jan 29;32(1):2444728. doi: 10.1080/26410397.2024.2444728 (PMC11884098; doi:10.1080/26410397.2024.2444728)
Supplement: Appendix A1. Focus Group Protocol. [file ZRHM_A_2444728_SM5170.docx]

**Appendix A.** **Interview Questions for Participants**

**Race and Gender**

1. In your own words, what comes to mind when you think of being a Black girl? Tell us what it means to you.

2. Let’s talk about how Black Girls are perceived or looked at by others. What are some words that come to mind?

a. How do you feel people view you in society?

3. There is a lot of talk about racism lately. We want to know in your own words, how do you define racism?

a. Have you experienced racism? How did it make you feel?

4. Do you think sexism exists? If so, have any of you experienced sexism or discrimination or been treated badly based on being a girl?

a. If yes, can you tell us about it?

5. Do you think racism and sexism is unique to Black girls?

a. Do you think Black girls are treated differently than other girls of color?

6. Based on how Black girls are viewed and treated, one of the things we want to understand is how this affects the behaviors that Black girls do that may be harmful to their health. When it comes to having sex as a teenager or using drugs for example, do you think the way Black girls are viewed affects the decisions they make around having sex? Why or why not?

a. What about using drugs? Do you think the way Black girls are viewed or treated causes them to use drugs? Why or why not?

**Sexual Health**

In the next section of our focus group, we’ll discuss sexual health.

1. Let's talk about HIV/AIDS, what do you all know about it?
   1. Do you think Black girls are at risk of getting HIV? Why or why not
   2. Where and how often do you hear information about HIV ?
      1. Probing question: can you give examples about the information you hear?
2. What about sexually transmitted infections?
   1. What do you know about them? Can you name a few?
3. Do you know anything about things to use for safe sex like condoms or even birth control pills? If so, where did you learn these things from?
4. How does your community address HIV and STI prevention for girls and women?
   1. Community can be neighborhood, family or school. However, you define community.
5. So as researchers, we can tell you that there is a close relationship between STIs, HIV, and drug use among girls. As we mentioned we want to create a prevention program for Black girls, by Black girls. So what would be a good way to prevent HIV/AIDS and STIs for Black girls?
   1. Probing question: what are some things that motivate people to protect themselves from HIV and sexually transmitted infections?

**Seeking Health Information**

1. According to research, young people get information about their health from various sources like social media, friends, schools, parents, what do you think is the best way to get this type of information? Which way do you prefer?
2. We want to create a prevention program for Black girls. What would you girls like to see in that program? What would you not like to see?
   1. Who should be leading the program?
   2. Any ideas on structure? Prefer in person? Social media? Text messaging?
   3. Who should be involved? Just girls? Or include Parents? Teachers? Mentors? Explain more what you would like their roles to be?

Of all the things we discussed, which did each of you think was the most important?

Is there anything that we have not discussed today or anything else that you would like to share before we end the focus group session?
